# Contrast‐enhanced ultrasonography characteristics of intrathoracic mass lesions in 36 dogs and 24 cats

**DOI:** 10.1111/vru.12698

**Published:** 2018-11-26

**Authors:** Thorsten Rick, Miriam Kleiter, Ilse Schwendenwein, Eberhard Ludewig, Martin Reifinger, Katharina M. Hittmair

**Affiliations:** ^1^ Department for Companion Animals and Horses University of Veterinary Medicine Vienna Austria; ^2^ Department for Pathobiology University of Veterinary Medicine Vienna Austria

**Keywords:** contrast‐enhanced ultrasound, mediastinal masses, pulmonary masses, small animals, thoracic ultrasonography

## Abstract

Contrast‐enhanced ultrasonography (CEUS) is increasingly available for veterinary patients, however limited studies describe the use of this method for characterizing intrathoracic mass lesions. The aim of this prospective, observational study was to describe CEUS enhancement patterns for intrathoracic mass lesions in a sample of cats and dogs. Sixty patients (36 dogs, 24 cats) were included. Standardized CEUS examinations were performed for 41 pulmonary masses (68%) and 19 mediastinal masses (32%). Final diagnosis was based on cytology and/or histopathology. Absolute time to enhancement (TTE) values were recorded for the intrathoracic mass lesions and spleen. The spleen was used as a reference parenchymal organ to calculate relative TTE (rTTE) values. Absolute TTE of the spleen and intrathoracic mass lesions differed for dogs and cats (*P* = 0.001). The rTTE values significantly differed between lesions of neoplastic versus non‐neoplastic origin (*P* = 0.004). The majority of neoplastic pulmonary masses were supplied by bronchial arteries (63%), while most nonneoplastic pulmonary masses were supplied by pulmonary arteries (78%). The sensitivity and specificity for detecting pulmonary neoplastic masses with rTTE were 63% and 78%, respectively. Enhancement patterns for mediastinal thymomas and lymphomas significantly differed (*P* = 0.002). Thymomas enhanced heterogeneously in a centripetal pattern (86%), whereas lymphomas typically enhanced uniformly in a centrifugal pattern (75%). Findings indicated that CEUS is a feasible method for characterizing intrathoracic mass lesions in dogs and cats, however, the diagnostic sensitivity for detecting neoplastic pulmonary masses was low.

**Abbreviations**
CEUS: contrast enhanced ultrasonography
MI: mechanical index
rTTE: relative time to enhancement
SD: standard deviation
TTE: time to enhancement
TTP: time to peak

## 1 INTRODUCTION

Mass lesions are defined as solitary lesions larger than 3 cm in size.^1^ Mass lesions in lung parenchyma can be caused by primary and metastatic neoplastic or nonneoplastic disorders. The majority of primary lung tumors in dogs and cats originate from airway epithelium.^2^ Adenocarcinoma, bronchoalveolar carcinoma, squamous cell carcinoma, and anaplastic carcinoma are the most common tumors found in the lung.^2^ Nonneoplastic pulmonary mass lesions include pneumonia, abscesses, granulomas, hematoma, cavitary lesions such as bullae, blebs, and cysts, and lung lobe torson.^1^ In the group of inflammatory mass lesions, pneumonia is most frequently diagnosed in dogs with lower respiratory tract disease while cats suffer most commonly from bronchial disease.^3^ The most common mass lesions in the mediastinum are lymphoma, thymoma, bronchogenic and idiopathic mediastinal cysts, ectopic thyroid, chemodectoma, and some rare neoplasms.^4^

Diagnosis can be challenging due to the diverse appearances of nonneoplastic and neoplastic intrathoracic mass lesions in dogs and cats. The use of contrast agents is a common tool in diagnostic imaging. Imaging modalities such as contrast enhanced CT, contrast enhanced MRI, perfusion scintigraphy, and angiography use vascularity patterns for characterization of intrathoracic masses. In recent years, ultrasonographic contrast agents have been used more frequently in veterinary medicine.^5^, ^6^, ^7^ However, there is little information on the use of CEUS in intrathoracic mass lesions in veterinary medicine.^8^, ^9^ Studies in human medicine were able to characterize different pulmonary masses with CEUS.^10^, ^11^, ^12^ Additionally, pulmonary lesions were found to differ in their arterial supply^10^ and vascularization of pulmonary neoplasia originates mainly from bronchial arteries.^11^ Vascularization is an important factor in tumor identification and the recognition of specific perfusion characteristics are of great diagnostic and therapeutic interest.^10^

The purposes of this study were to assess the feasibility of CEUS as a method for characterizing pulmonary and mediastinal mass lesions in dogs and cats, and to determine whether CEUS could distinguish neoplastic from nonneoplastic pulmonary and mediastinal mass lesions. The hypothesis was that nonneoplastic pulmonary mass lesions would differ from neoplastic mass lesions regarding their vascular supply, with neoplastic mass lesions supplied mainly by systemic bronchial arteries. For mediastinal mass lesions, the hypothesis was that, due to the different angioarchitecture, lymphoma and thymoma tumor types would differ in dynamic enhancement characteristics.

## 2 MATERIAL AND METHODS

All procedures were discussed and approved by the institutional ethics and animal welfare committee in accordance with good scientific practice guidelines and national legislation. Dogs and cats were included in this prospective, observational study when thoracic radiographs showed an intrathoracic mass of at least 3 cm in size and which was available for ultrasound guided sampling during the period of October 2014 to January 2017. Sample size was based on convenience sampling. Exclusion criteria were nondiagnostic samples and therefore no final diagnosis of the mass was performed. The decision for inclusion or exclusion of cases as well as for recording imaging and pathologic findings were made after consensus assessment by two university hospital radiologists (T.R. and K.H.) with one of them experienced in contrast ultrasonography (K.H.). The same authors performed all imaging procedures.

Ultrasonography of the mass was performed and the origin of the lesion was determined to be either pulmonary or mediastinal. Each patient was examined with an 8–5 MHz curved array transducer (iU22 Philips, Bothell, WA). The ultrasound examination was carried out with special emphasis to localization, echogenicity, texture, and contour of the mass, and amount of free fluid in the thoracic cavity. The CEUS procedures and diagnostic biopsies were obtained either under sedation with butorphanol alone (0.1–0.3 mg/kg, Alvegesic^®^, Alvetra und Werfft GmbH, Vienna, Austria) or under short anesthesia with butorphanol and propofol (Propofol, Fresenius Kabi Austria GmbH, Graz, Austria) if needed. The CEUS examination of the intrathoracic mass was performed first. After a break of 5 min, a second contrast medium injection was performed for the CEUS examination of the spleen. A convex 5–1‐MHz transducer was used with machine settings comparable in both CEUS examinations of the intrathoracic mass and spleen. The CEUS examination was performed with a low mechanical index (MI) setting (0.05–0.11). The focus was set outside the region of interest to not influence the MI. The settings were kept constant for all patients.

A total of 1 to 1.5 mL (1 mL < 20 kg; 1.5 mL > 20 kg) of contrast medium (SonoVue^®^, Bracco International B.V., Amsterdam, the Netherlands) was injected through a peripheral venous catheter placed in the cephalic vein. The catheter was equipped with a three‐way valve and a tube extension. Each contrast injection was followed by a 5 mL saline flush (0.9% NaCl) through the cross connection of the three‐way‐valve.

After injection of ultrasonographic contrast agent, enhancement patterns were recorded for 120 s. The videos were analyzed using specific software (QLab, Philips, Andover, USA) installed on the ultrasound machine. All measurements of contrast enhancement in the mass were compared to those in the spleen as a parenchymal, systemically perfused reference organ. In two dogs, the liver was used due to previous splenectomy as a result of a ruptured hemangiosarcoma in one dog, and because of splenic nodules of unknown origin in the other.

**Figure 1:**
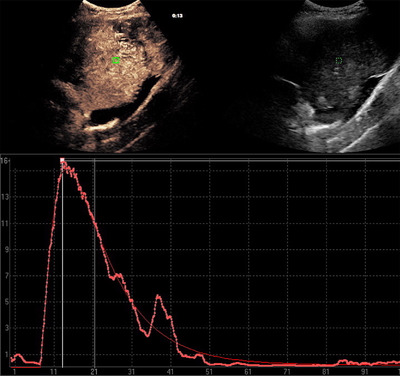
Region of interest placement within the lesion. A standardized 5 × 5 mm region of interest was placed at a representative region within the lesion where enhancement was most obvious and no cysts or large vessels were detected. On the right side is the B‐mode image, on the left the side the contrast study. The graph is a signal intensity (*y*‐axis; in decibel) to time (*x*‐axis; in seconds) curve [Color figure can be viewed at wileyonlinelibrary.com]

Standardized 5 × 5 mm regions of interest were placed within the lesion where enhancement was most obvious and no large vessels or cysts were located (Figure 1). Signal intensity time curves were generated and used for quantitative and qualitative measurements. Quantitative time parameters of circulation velocity such as absolute TTE (time from injection to enhancement), time to peak (TTP, time from injection to maximum intensity), wash‐in time, wash‐out time, and maximum contrast intensity in decibel (dB) were evaluated. Wash‐in reflected the time from the start of enhancement to peak intensity and wash‐out, the time when intensity decreased to 50% of peak intensity. Qualitative parameters, such as uniformity of enhancement, distribution and perfusion of enhancement from central or peripheral, and presence of nonenhancing regions within the lesion were recorded.

For pulmonary lesions, the type of vascularization (pulmonary or bronchial arterial) was determined by comparing the absolute TTE in the lesion to that in the systemically perfused spleen. From this ratio, the relative TTE (rTTE) was calculated (TTE of the lesion/TTE of the spleen = rTTE). The rTTE was defined as “early” if enhancement of the mass was observed before enhancement of the spleen, and “delayed” if it was observed at the same time or later than the spleen. An early rTTE had values < 1 and affected masses were defined as pulmonary arterial vascularized and delayed rTTE had values equal to or higher than 1 and masses were defined as bronchial arterial vascularized.

In all patients a fine needle aspiration cytology and/or histology was performed. The type of sampling was determined by the size and location of the lesion and the general condition of the patient.

Descriptive data were depicted in a contingency table. Patients were divided into seven groups regarding their cytology and/or histology results. Differences between groups in absolute and relative TTE, TTP, wash‐in, wash‐out, and maximum intensity were analyzed using general linear models followed by post hoc tests using Bonferroni's alpha correction procedure. The discrete data of the qualitative CEUS measurements, such as type of vascularization (pulmonary arterial or bronchial arterial), presence of nonenhancing regions (yes or no), and origin of enhancement (central or peripheral) were depicted in contingency tables and analyzed by using Fisher's exact test. Regarding the type of vascularization in pulmonary masses, sensitivity and specificity for detecting neoplastic transformation with the value rTTE were calculated. For all statistical tests, a *P*‐value below 5% (*P* < 0.05) was considered as statistically significant.

Group seven contains rare masses in both the lung and mediastinum, which were found once and were therefore regarded as single values. No statistical analysis was done between group seven and the other groups. All statistical tests were selected and analyzed by a professional university statistician using IBM SPSS Statistics v24 (IBM Corporation, Armonk, NY).

## 3 RESULTS

Sixty animals (24 cats, 36 dogs) met inclusion criteria for the study. Of the 24 cats, 13 were neutered males (54%) and 11 were spayed females (46%). Seventeen dogs were male (47%, seven neutered, 10 intact) and 19 were female (53%, 13 spayed, six intact). The mean age of the feline patients was 11 years (range: 1–17 years), and that of the dogs was 10 years (range: 1–15 years). The mean weight in cats was 4.3 kg (range: 2–7 kg) and in dogs, 23 kg (range: 5–50 kg). The group of cats included Domestic Shorthairs (*n* = 21), and one of each Abessiner, Burma, and Russian Blue. Dog breeds included mixed‐breed dogs (7), Golden Retriever (4), Boxer (2), Cavalier King Charles Spaniel (2), German Pinscher (2), and one of each of the following breeds: American Staffordshire Terrier, Bandog, Beagle, Bernese Mountain Dog, Cocker Spaniel, Collie, German Shepherd, German Shorthair, English Springer Spaniel, Flat Coated Retriever, Jack Russel Terrier, Labrador Retriever, Magyar Vizsla, Pug, Rottweiler, Shiba Inu, Shih Tzu, Tibet Terrier, and Berger Blanc Suisse.

Intrathoracic mass lesions were found on radiographs in all but one case, which was discovered during thoracic CT.

In all cases, fine needle aspiration cytology and/or histology was performed. Fine needle aspiration was performed in 54 of 60 patients (90%) and histology in 19 of 60 cases (32%). Both procedures were applied in 13 of 60 cases (22%) and agreed in 11/13 cases (85%). In cases of disagreement, the histopathologic result was used. Of the 41 pulmonary masses, 14 (32%, five cats, nine dogs) were diagnosed as adenocarcinomas, seven (17%, five dogs, two cats) were undifferentiated carcinomas, nine (22%, five cats, four dogs) were sarcomas, one (2%, one dog) lymphoma, and 10 (24%, five dogs, five cats) were nonneoplastic mass lesions. The group of nonneoplastic masses included nine inflammatory consolidations and one lung lobe torsion.

Of the 19 mediastinal masses seven (37%, three cats, four dogs) were diagnosed as thymomas, eight (42%, four cats, four dogs) as malignant lymphomas, and one of each of the following tumors: malignant nerve sheath tumor, hemangiosarcoma, tracheal B‐cell lymphoma, and melanoma.

The patients were assigned into seven groups according to their cytology or histology results (Table 1). Groups 1 to 4 represent pulmonary neoplastic and nonneoplastic masses (adenocarcinomas, undifferentiated carcinomas, sarcomas, and inflammatory mass lesions). The group of nonneoplastic masses contains inflammatory mass lesions such as pneumonia (7) , bronchopneumonia (1) and one abscess (1). Groups 5 and 6 represent mediastinal masses (thymoma and mediastinal lymphoma). Group 7 contains masses of pulmonary and mediastinal origin that occurred once each and were considered individually (Table 2).

**Table 1:** Groups of intrathoracic mass lesions examined with contrast enhanced ultrasonography. Patients were grouped according to their cytology or histology results into seven groups

Groups of pathologies					
			Species		
			Dogs	Cats	Total
Group	1. Adenocarcinoma	Number	9	5	14
		Percent	64.3%	35.7%	100.0%
	2. Undifferentiated carcinoma	Number	5	2	7
		Percent	71.4%	28.6%	100.0%
	3. Sarcoma	Number	4	5	9
		Percent	44.4%	55.6%	100.0%
	4. Nonneoplastic/inflammatory	Number	6	3	9
		Percent	66.7%	33.3%	100.0%
	5. Mediastinal thymoma	Number	4	3	7
		Percent	57.1%	42.9%	100.0%
	6. Mediastinal lymphoma	Number	3	5	8
		Percent	37.5%	62.5%	100.0%
	7. Others	Number	5	1	6
		Percent	83.3%	16.7%	100.0%
Total		Number	36	24	60
		Percent	60.0%	40.0%	100.0%

**Table 2:** Contrast enhanced ultrasonography characteristics of six other intrathoracic masses

	Absolute TTE (s)	Relative TTE	TTP (s)	Wash‐in time (s)	Wash‐out time (s)	Maximum contrast intensity (dB)	Homogeneity of enhancement
Generalized pulmonary lymphoma	4	early	22	11	98	14	homogeneous
Pulmonary lung lobe torsion	0	0	0	0	0	0	no enhancement
Mediastinal nerve sheath tumor	16	delayed	36	17	25	4	heterogeneous
Mediastinal hemangiosarcoma	8	delayed	20	7	47	12	homogeneous
Mediastinal melanoma	12	delayed	24	7	39	14	heterogeneous
Tracheal lymphoma	3	delayed	13	7	15	15	heterogeneous

Abbreviations: dB, decib; s, seconds; TTE, time to enhancement; TTP, time to peak.

There was no significant difference regarding wash‐in, wash‐out time, TTP, and maximum intensity between the groups. In four patients, no contrast enhancement was observed. Two were adenocarcinomas, one was a lung lobe torsion, and one chronic pneumonia.

**Figure 2:**
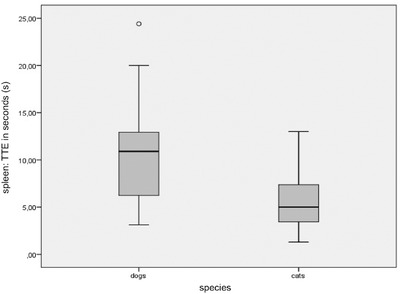
Time to enhancement of the spleen in dogs and cats. Absolute TTE of the spleen represented systemic circulation time and differed significantly in dogs and cats. TTE, time to enhancement

All continuous variables in the statistical analysis were normally distributed as assessed by a Shapiro‐Wilk test. The absolute TTE of the spleen represented systemic circulation time and differed significantly in both species (*P* = 0.001; Figure 2). In cats, mean TTE was 5.7 s (range 1 – 13 s). In dogs, mean TTE of the spleen was 10.5 s (range: 3–24 s). Absolute TTE of all intrathoracic masses ranged from 1 to 28 s in dogs (mean 9.4 s, standard deviation [SD] 6.5 s) and from 1 to 12 s in cats (mean 4.6 s, SD 2.8 s). This was significantly different between these two species (*P* = 0.002).

**Figure 3:**
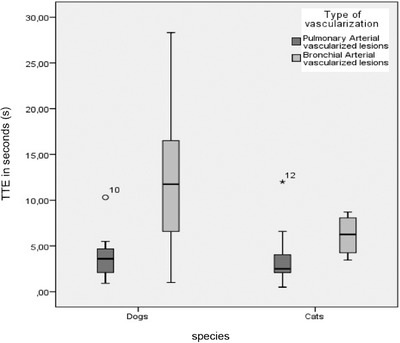
TTE in dogs and cats. Time to enhancement in seconds in pulmonary arterial vascularized (early rTTE) and bronchial arterial vascularized (delayed rTTE) masses in dogs and cats. Bronchial arterial vascularized masses in cats enhance significantly earlier than those in dogs. The difference in TTE of bronchial arterial vascularized masses between these species was statistically significant. The TTE values of bronchial arterial vascularized masses vary within a wider range in dogs compared to cats due to the greater variety of sizes and breeds in this species. There was no significant difference in pulmonary arterial vascularized masses between dogs and cats. rTTE, relative time to enhancement; TTE, time to enhancement

The absolute TTE of the mass was compared to the absolute TTE of the spleen to calculate rTTE for each patient. Pulmonary arterial vascularized masses with an early rTTE had absolute TTE values of 1–12 s (mean: 4.5 s, SD 3.2 s) in both species. In cats, TTE of these masses ranged from 1 to 12 s (mean: 3.64 s, SD 3.1 s) and in dogs from 1 to 11 s (mean: 5.2 s, SD 3.1 s). Bronchial arterial vascularized masses with a delayed rTTE had an absolute TTE of 1–28 seconds (mean 9.8 s, SD 6.3 s) in all patients (Figure 3). In cats, TTE of these lesions ranged from 2 to 9 s (mean: 5.42 s, SD 2.1 s) and in dogs from 1 to 28 s (mean: 12.2 s, SD 6.5 s). This difference was statistically significant (*P* = 0.002).

**Figure 4:**
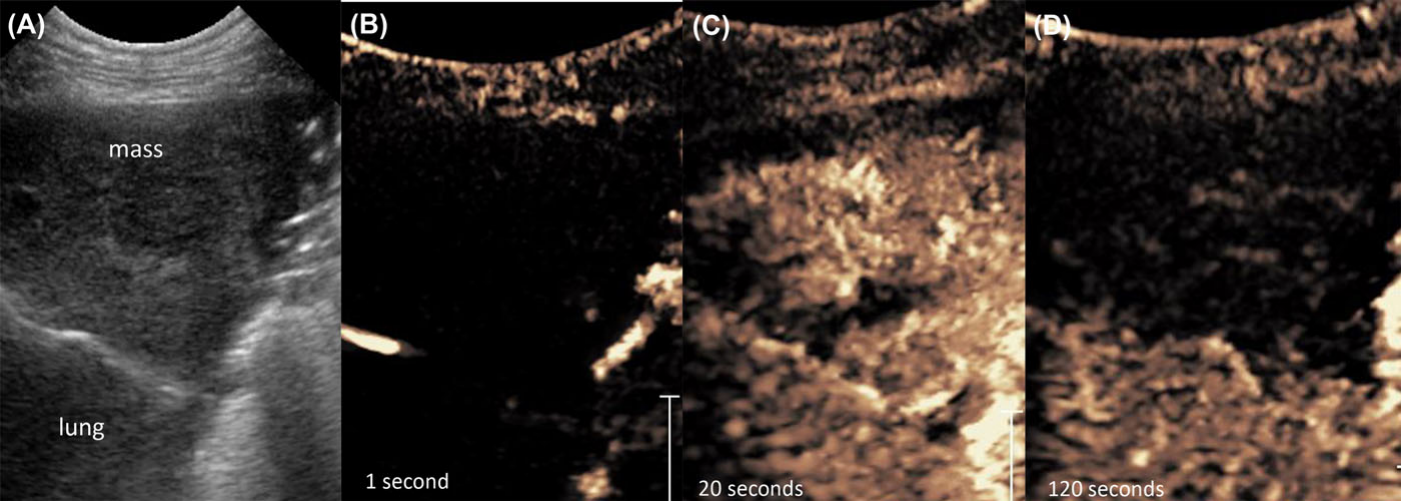
Pulmonary adenocarcinoma. Eight‐year‐old female neutered German Pinscher with an adenocarcinoma in the left caudal lung lobe. A, B‐mode sonography shows a triangular, hypoechoic, pleural based mass‐lesion. B and C, Contrast enhanced ultrasonography shows a delayed enhancement at the same time as the spleen. D, After 120 s no enhancement was visible within the mass [Color figure can be viewed at wileyonlinelibrary.com]

Benign and malignant pulmonary lesions varied significantly regarding rTTE (*P* = 0.004). In pulmonary masses an early rTTE was observed in 16 cases and a delayed rTTE in 20 cases (Figure 4). Most of the nonneoplastic, inflammatory cases (78%) had an early rTTE, while only one patient with an inflammatory lung mass lesion had a delayed rTTE (11%). The remaining case did not show enhancement (11%). About two‐thirds of all pulmonary neoplastic lesions (63%) showed a delayed rTTE and one third (30%) had an early rTTE (Table 3). For the differentiation of non‐neoplastic and neoplastic pulmonary changes regarding rTTE, a sensitivity (ie, the probability that a delayed rTTE will indicate neoplastic transformation) of 63% (95% CI, 45–80%) and a specificity (ie, the propability that an early rTTE will indicate no neoplastic infiltration) of 78% (95% CI, 51–100% ) was calculated.

**Table 3:** Type of vascularization: Pulmonary masses

Type of vascularization: Pulmonary masses (rTTE)						
	Type of vascularization					
			No enhancement	PA (early rTTE)	BA (delayed rTTE)	Total
Pulmonary:	Adenocarcinoma	Number	2	5	7	14
		Percent	14.0%	36.0%	50.0%	100.0%
	Undifferentiated carcinoma	Number	0	3	4	7
		Percent	0.0%	43.0%	57.0%	100.0%
	Sarcoma	Number	0	1	8^a^	9
		Percent	0.0%	11.1%	88.9%	100.0%
	Nonneoplastic/ inflammatory	Number	1	7^a^	1^a^	9
		Percent	11.1%	77.8%	11.1%	100.0%
Total		Number	3	16	20	39
		Percent	7.7%	41.0%	51.3%	100.0%

*Notes*. Time to enhancement (TTE) data in subgroups of intrapulmonary lesions in relation to aetiology. Vascularization was defined as pulmonary arterial (PA) when TTE within the lesion was observed before enhancement of the spleen (early rTTE) and as bronchial arterial (BA) when it was observed at the same time or later than the spleen (delayed rTTE). Benign and malignant pulmonary lesions varied significantly regarding rTTE (*P* = 0.004). Most nonneoplastic, inflammatory cases (78%) had an early rTTE, while only one patient with an inflammatory lung mass lesion had a delayed rTTE (11%). A majority of pulmonary neoplastic lesions (63%) showed a delayed rTTE and one third (30%) had an early rTTE.

a Statistically significant difference.

Abbreviations: BA, bronchial arterial; PA, pulmonary arterial; rTTE, relative time to enhancement.

**Figure 5:**
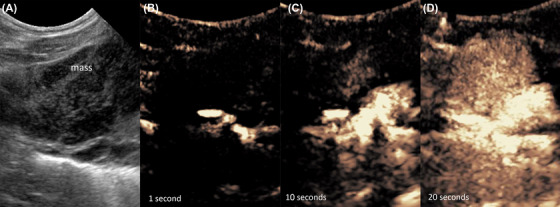
Mediastinal Lymphoma. Twelve‐year‐old, castrated male Domestic Shorthaired cat with a precardial, mediastinal mass lesion. Mediastinal lymphoma was confirmed by cytology. A, B‐mode sonography shows a round mass of mixed echogenicity. B–D, Contrast enhanced ultrasonography shows an homogeneous enhancement after about 10 s originating from central and spreading to the periphery of the lesion [Color figure can be viewed at wileyonlinelibrary.com]

**Figure 6:**
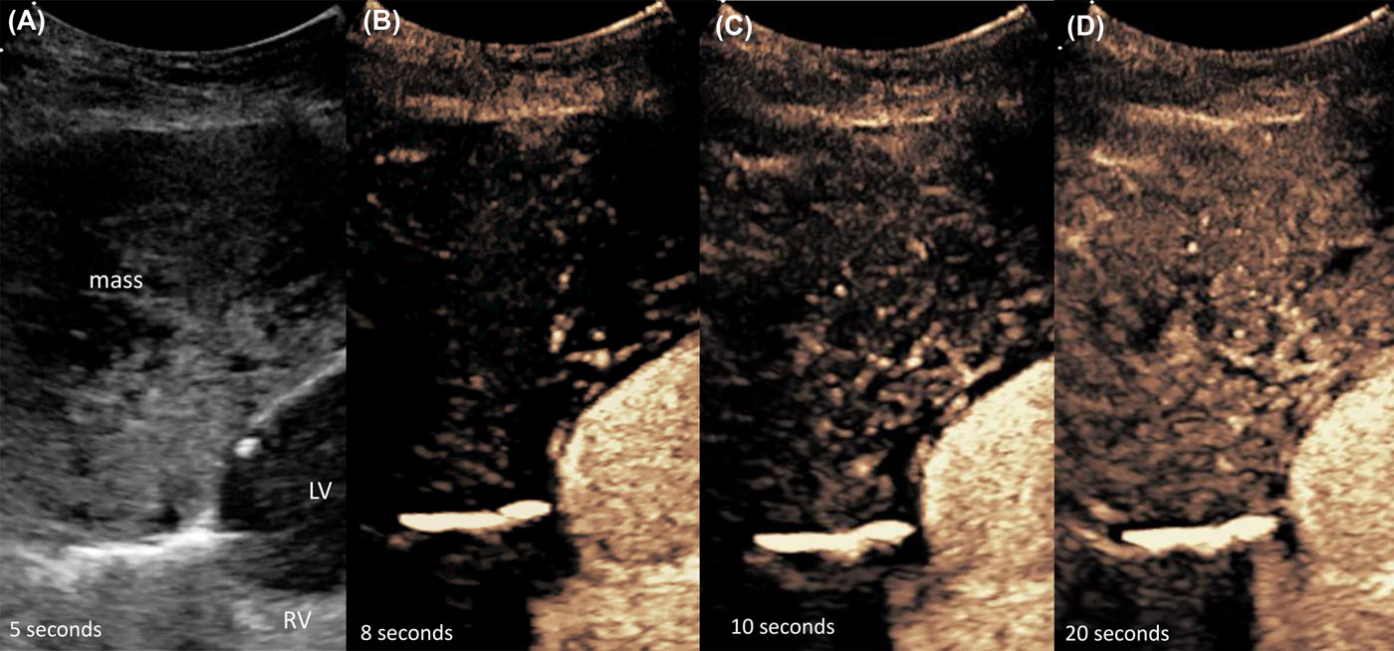
Thymoma. Thirteen‐year‐old, male castrated Golden Retriever with a mediastinal thymoma. A, The mediastinal lesion and both heart chambers are depicted. B and C, Contrast medium perfuses the mass after passing the RV‐ and LV‐heart chamber from peripheral to central. C, At peak intensity, there are multiple nonenhancing regions visible in thymomas. LV, left ventricle; RV, right ventricle [Color figure can be viewed at wileyonlinelibrary.com]

Seven thymomas and eight mediastinal lymphomas were found. A heterogeneous and cystic parenchyma was found in three of seven (43%) thymomas during B‐mode ultrasonography, while four of seven (57%) thymomas showed nonenhancing areas with CEUS. Lymphomas enhanced uniformly in all cases (*P* = 0.009; Table 4). Another significant difference between thymoma and lymphoma was the distribution of enhancement. In six of seven (86%) thymomas, an enhancement from peripheral to central was observed, which showed a significant difference to mediastinal lymphomas in which enhancement was typically from central to peripheral (*P* = 0.016; Table 4; Figures 5 and 6).

**Table 4:** Contrast enhancement in thymoma and mediastinal lymphoma

			Nonenhancing areas			Enhancement		
			No	Yes	Total	From central	From peripheral	Total
Group	Thymoma	Number	3	4^a^	7	1^a^	6	7
		Percent	42.9%	57.1%	100.0%	14.3%	85.7%	100.0%
	Lymphoma	Number	8	0^a^	8	6^a^	2	8
		Percent	100.0%	0.0%	100.0%	75.0%	25.0%	100.0%
Total		Number	11	4	15	7	8	15
		Percent	73.3%	26.7%	100.0%	46.7%	53.3%	100.0%

*Notes*. Contrast enhanced sonographic characteristics of subgroups of mediastinal masses. Thymomas show significantly more frequently non‐enhancing areas after contrast administration than mediastinal lymphoma and tend to enhance from peripheral to central while most lymphomas enhance from central to peripheral.

a Statistically significant difference.

## 4 DISCUSSION

Findings from the current study supported our hypothesis that CEUS enhancement and vascularization patterns are different in a portion of neoplastic and nonneoplastic pulmonary and mediastinal mass lesions. In pulmonary masses, static enhancement patterns have more impact for decision making than dynamic enhancement patterns.

**Figure 7:**
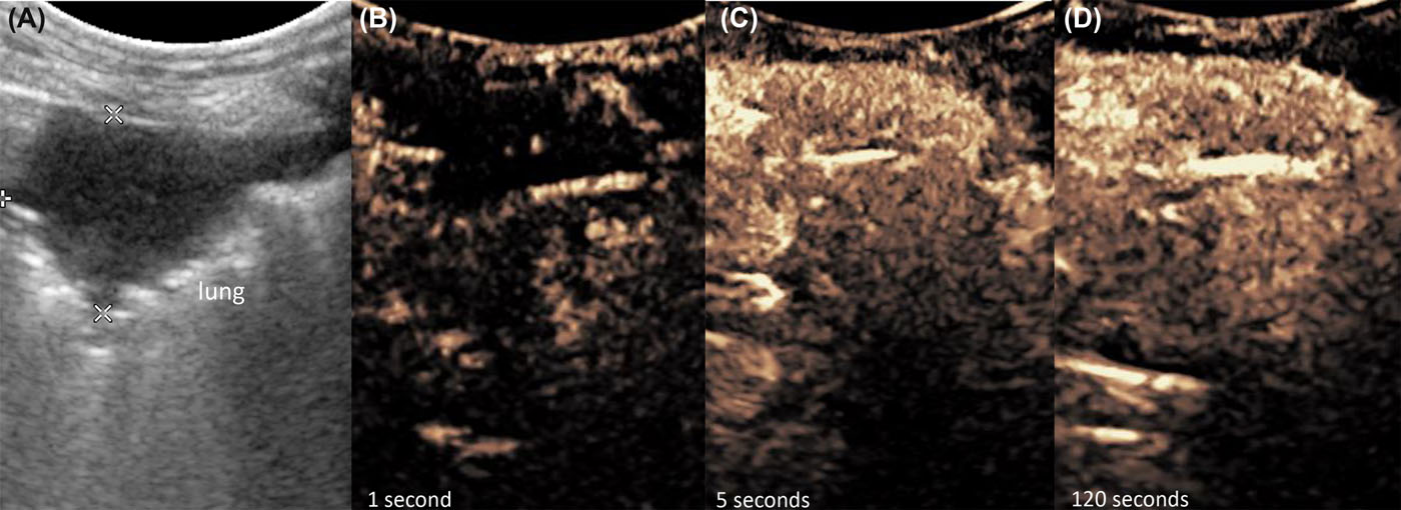
Pulmonary inflammatory lesion. Nine‐year‐old, neutered female mixed breed dog with an inflammatory lung infiltration. A, B‐mode sonography shows a triangular, hypoechoic lung consolidation. B, Contrast enhanced ultrasonography shows an early rTTE after a few seconds, there was no enhancement in the spleen at that time, which is indicative for a pulmonary arterial supply in that lesion. C, At peak intensity, there is a homogeneous marked enhancement. The contrast remains in the lesion for at least 120 s [Color figure can be viewed at wileyonlinelibrary.com]

The exact time of contrast enhancement within a lesion depends on the patient's individual circulation time, which is why a systemic organ was used as reference in each patient. A majority of malignant pulmonary lesions showed a delayed rTTE and therefore bronchial arterial vascularization (Figure 4) compared to benign pulmonary lesions, which were supplied mainly by pulmonary arteries (Figure 7).

The calculation of sensitivity and specificity of 63% and 78%, respectively, for detecting a neoplastic pulmonary mass lesion based on the type of vascularization, demonstrate the relatively high number of malignant mass lesions vascularized by pulmonary arteries and therefore high number of false negative results. Determination of a systemically perfused mass lesion with a delayed rTTE provides a better characterization of the contrast enhancement and a more accurate diagnosis. These findings were consistent with studies in human medicine where primary pulmonary malignancies have a prolonged TTE and therefore probably a bronchial arterial circulation, while normal lung parenchyma shows a short TTE with pulmonary arterial vascularization.^11^, ^12^ The pulmonary blood supply is fed by two systems. Pulmonary arteries are responsible for pulmonary gas exchange and carry deoxygenated blood from the right heart chamber to the pulmonary capillaries. The bronchial arteries provide nutrition for the lung and arise from the aortic arch and intercostal arteries.^10^

Pulmonary and mediastinal masses were examined, with a systemically perfused organ as a self‐reference in every patient. Numerous factors can influence the wash‐in and perfusion of contrast in an organ, including type and location of the venous catheter, heart rate, cardiac output, as well as existing lung diseases. To better discriminate between pulmonary arterial and systemic vascularization patterns, it is useful to use a reference organ with a systemic vascular supply such as the liver or spleen. Contrast enhancement in a lung lesion prior to systemic enhancement characterizes pulmonary arterial vascularization, while contrast enhancement coinciding with systemic enhancement shows a systemic or bronchial arterial vascular supply of the lung lesion.^13^ Pulmonary arteries originate from the right heart chamber, and masses that are supplied by these arteries enhance earlier than masses supplied by bronchial arteries stemming from systemic circulation (Figure 7).

Studiesin human medicine have shown that the nutritional supply in lung tumors is due to neovascularization into an existing blood supply, often affecting bronchial arteries.^10^, ^11^, ^12^, ^13^, ^14^ Benign, inflammatory lesions in this study were supplied by pulmonary arteries and enhanced before the systemically perfused spleen. With pneumonia or bronchopneumonia, inflammatory cells migrate to the affected region and pulmonary arteries dilate, resulting in a bright, short CEUS signal.^10^ These underlying considerations could explain the early rTTE in 78% of benign, inflammatory pulmonary lesions. In one patient suffering from an inflammatory lesion, a delayed rTTE was observed, suggestive of a change from pulmonary to bronchial arterial supply. Angiographic studies in human medicine determined that some inflammatory cavitary lung lesions such as lung cysts, parapneumonic abscesses, and liquified pneumonia are occasionally supplied by bronchial arteries.^14^ Some of the malignant lesions in this study also had an early rTTE, indicative of pulmonary arterial vascularization. These findings are comparable to human medicine, in which studies have shown that some adenocarcinoma and bronchio‐alveolar carcinoma subtypes are perfused by neovascularization originating from pulmonary arteries as well.^15^ Furthermore, tumors may be able to grow without neovascularization if they find an appropriate “vascular bed”.^15^ Another important factor is the size of the tumor. Due to rapid growth in some tumor types, the bronchial arterial supply is small and additional neovascularization originates from pulmonary arteries.^11^

There were no significant differences between groups of pulmonary or mediastinal lesions regarding the quantitative measurements TTP, wash‐in time, wash‐out time, and maximum signal intensity. In mediastinal masses, dynamic enhancement patterns were useful to differentiate between thymoma and lymphoma. Mediastinal thymomas showed nonperfused areas in more than half of the cases, similar to previous studies in human and veterinary medicine .^16^, ^17^ In this study, only one thymoma presented homogeneously on grayscale ultrasound and demonstrated nonenhancing areas with CEUS, which means that grayscale ultrasonography is sufficient to detect this characteristic. In this study, nonperfused areas were absent in mediastinal lymphomas . A significant difference was noticed in the distribution of enhancement. In mediastinal lymphomas, enhancement was centrifugal. The perfusion of lymph nodes is maintained over a central hilus vessel with numerous lymphatic sinuses and appears as a bright hyperechoic central region.^18^ The angioarchitecture of lymphomatous lymph nodes has been described in veterinary medicine.^19^ Contrast enhanced ultrasonography can display vascular changes in malignant lymph nodes with the normal hilus vessel displaced or not visible.^19^ The angioarchitecture of thymomas is based on more superficial vessels, which explains the perfusion from peripheral to central.^20^

Enhancement in cats was significantly different from enhancement in dogs (*P* = 0.001), which was likely explained by the different cardiovascular status and circulation times in these species. Cats had a significantly earlier absolute TTE (mean 5.7 s) than dogs (mean 10.5 s). The values in dogs were slightly higher than previously reported in sedated canine patients.^21^ Rossi et al. published an arrival time of contrast in the spleen of 8.77 ± 1.16 s in non‐sedated dogs and 7.6 ± 1.16 s in butorphanol‐sedated dogs.^21^ Reasons for the difference are unclear due to the different study designs and amount of sedation.

Cats have a significantly shorter absolute TTE (mean 4.64 s) in intrathoracic mass lesions than dogs (mean 9.45 s) and an individual reference organ should be used in each patient.

Five masses showed no contrast enhancement. With lung lobe torsion, the pulmonary blood flow is compromised and leads to varying contrast enhancement depending on the degree of torsion.^8^ Another nonenhancing, nonneoplastic mass lesion was a chronic inflammatory process. Studies in human medicine report that pulmonary arteries react to hypoxia by vasoconstriction called Euler‐Liljestrand reflex and may be the cause for the nonenhancing region.^22^

In the present study, fine needle aspiration of the mass lesion was used to determine the final diagnosis and was performed in 90.1% of cases. Histology was carried out in 32% of the cases. Both modalities agreed in 11 of 13 (84.6%) cases, which is similar to previous studies in veterinary medicine with agreements of 82%.^23^ Furthermore, the sensitivity and specificity of FNA cytology for the diagnosis of neoplasia in the lung has been reported to be 77% and 100%, respectively.^23^

Enhancement should be compared to a systemically perfused organ where an actual hemodynamic situation is represented. Most of the patients were sedated with a small dose of butorphanol. The influence of anesthesia on CEUS has been studied and reports have shown that certain anesthetics have a significant influence on contrast enhancement in dogs and cats.^21^, ^24^ TTE was reportedly delayed in anesthetized cats and dogs, and anesthesia may increase heterogeneity in the spleen of cats.^21^ Butorphanol sedation and propofol anaesthesia have been found to not influence blood volume or CEUS parameters.^21^, ^25^

The main limitation of this study was the relatively heterogeneous group of patients with different species and different cardiovascular conditions and the small numbers of dogs and cats within each subgroup. Use of the spleen as self‐reference were useful to classify enhancement patterns in each patient.

All neoplastic lung masses were considered primary lung tumors. However, it cannot be completely ruled out that some of the masses diagnosed as primary lung tumors may represent metastases. Studies in human medicine do not indicate a significant difference between vascularization of primary and metastatic lung masses.^11^

In conclusion, CEUS of intrathoracic mass lesions provided useful information to characterize pulmonary and mediastinal mass lesions in this sample of dogs and cats. However, the test had low sensitivity and specificity for differentiating neoplastic pulmonary masses and therefore findings in individual clinical patients should be interpreted with caution.

## LIST OF AUTHOR CONTRIBUTIONS

### Category 1

(a) Conception and Design: Hittmair KM
(b) Acquisition of Data: Rick T, Hittmair KM, Schwendenwein I, Reifinger M
(c) Analysis and Interpretation of Data: Rick T, Hittmair KM, Schwendenwein I, Ludewig E, Reifinger M

### Category 2

(a) Drafting the Article: Rick T, Hittmair KM
(b) Revising the Article for Intellectual Content: Hittmair KM, Kleiter M, Schwendenwein I, Ludewig E, Reifinger M

### Category 3

(a) Final Approval of the Completed Article: Rick T, Hittmair KM, Kleiter M, Schwendenwein I, Ludewig E, Reifinger M
